# The influence of adjacent anatomical context in tooth staging: observational study of non-cropped vs. cropped radiographs for dental age estimation

**DOI:** 10.1007/s00414-026-03929-4

**Published:** 2026-07-23

**Authors:** Nikolaos Angelakopoulos, Rizky Merdietio Boedi, Stefano De Luca, Israel Soriano Vázquez, Ademir Franco

**Affiliations:** 1 Department of Orthodontics and Dentofacial Orthopedics, University of Bern, Bern, Switzerland; 2 Department of Forensic Dentistry, Faculdade São Leopoldo Mandic, Campinas, Brazil; 3 Department of Dentistry, Faculty of Medicine, Universitas Diponegoro, Semarang, Indonesia; 4 Department of Forensic Medicine, Sechenov First Moscow State Medical University, Moscow, Russia; 5 Area of Physical Anthropology, Department of Organisms and Systems Biology, Faculty of Biology, University of Oviedo, Oviedo, Spain; 6 Forensic Odontologist, independent researcher, Mexico City, Mexico

**Keywords:** Age estimation, Demirjian system, Dental age, Reliability, Forensic dentistry, Image cropping, Inter-observer agreement, Regions of interest

## Abstract

Dental age estimation (DAE) is applicable to forensic/legal and clinical contexts, with the Demirjian staging system being among the most common techniques. However, inter- and intra-observer variability can affect DAE reliability, particularly when evaluating panoramic radiographs influenced by magnification, distortion, and anatomical superimposition. In the staging process, consciously or unconsciously, experts analyze dental development within the available peripheral anatomic features of the tooth and its peripheral region. This study investigated whether isolating teeth as regions of interest (cropped images) influences the reproducibility of Demirjian’s stage allocation. An observational cross-sectional study was conducted using 301 panoramic radiographs from subjects aged 7–14 years. Three experienced forensic odontologists independently staged all seven left mandibular teeth (#31–#37) and, when present, also the left mandibular third molar (#38) using the Demirjian’s staging system. Each tooth was evaluated twice, two weeks apart, in both full panoramic radiograph and cropped formats. Inter- and intra-observer agreement was assessed using kappa statistics. Intra-observer agreement was above 0.81. Cropped images resulted in only marginal changes, with a slight decrease in stage reproducibility (Δ − 0.017 and − 0.023). Inter-observer agreement was nearly identical between non-cropped (AC2 = 0.985) and cropped images (AC2 = 0.986). A slightly lower proportion of cropped images was assessable by all observers (90.7% vs. 91.7%). Isolating teeth as regions of interest did not meaningfully reduce reproducibility and only small intra-observer decreases were observed and concentrated in the anterior teeth. These findings indicate that, among experienced observers, cropping does not degrade the reliability of Demirjian stage allocation. Future DAE staging systems should consider being more descriptive, including not only tooth-related developmental details, but surrounding information, such as the distance between the analyzed tooth and the occlusal plane and its relationship with adjacent developed teeth.

## Introduction

Dental age estimation (DAE) is employed in daily forensic/legal practice, particularly in the forensic age estimation of asylum seekers [1, 2], the verification of reported minor status in legal proceedings [1], and the ascertainment of eligibility for age-restricted competitive sports [2–4]. Additionally, within anthropological research, DAE serves as a fundamental approach for achieving accurate age data essential to population-based investigations [5]. These applications highlight its relevance in situations where reliable documentation of chronological age is unavailable [6].

The process of DAE is based on the systematic analysis of dental variables that show consistent and predictable age-related changes [7–9]. Researchers and practitioners utilize well-established methodologies to obtain an estimated dental age [10]. However, these methodologies are prone to observer variation, which refers to inconsistencies within or between observers applying the same method [11]. The use of developmental stages of teeth for DAE is widely regarded as more accurate and reliable than assessment based solely on tooth eruption [12]. Several methods for estimating dental age via the radiographic analysis of dental development have been proposed, including Nolla’s [13], Willems’s [10], and the London Atlas [14].

The Demirjian staging system [15] is one of the most widely adopted in DAE, owing to its clearly defined developmental stages and its reputation for methodological simplicity and ease of application [16]. Nevertheless, the method is also affected by limitations in inter- and intra-observer consistency [17]. Inconsistencies often arise when observers interpret subtle differences in root length, apical development, or crown–root proportions.

One major source of variability in the Demirjian staging system [15] arises from the use of panoramic radiographs. This type of image is susceptible to magnification gradients, geometric distortion, and superimposition of anatomical structures, all of which can affect the apparent morphology of teeth and, consequently, the stage allocation process and the accuracy of DAE [18]. The position of the tooth itself, including its inherent angulation in the arch is also a factor. For example, when root length is less than the crown length, it may appear longer due to distortion, leading to an incorrect allocation into a more advanced stage. Conversely, a root that has completed its development without reaching apical closure might appear shorter due to buccolingual tooth angulation, which can lead to an incorrect allocation into an earlier stage [19, 20]. These factors can potentially lower staging reliability and can lead to considerable error in the real-life application of the methodology.

An additional, frequently overlooked factor contributing to staging variability is the influence of surrounding anatomical structures on the perceived morphology of the tooth [21]. When the entire panoramic radiograph is available, expert’s judgment may be influenced, consciously or unconsciously, by visual cues unrelated to the Demirjian criteria for dental development [15]. In other words, these cues are not stage-related and encompass peripheral details, such as eruption status (distance from tooth occlusal surface to the occlusal plane), proximity to adjacent teeth, and relationship with the surrounding alveolar bone. Such contextual cues may introduce cognitive bias, prompting the examiner to assign a developmental stage based on apparent eruption or relative position, even if the original staging technique does not advocate such a procedure [22].

Prior research has indicated that anatomical positional cues, such as relative tooth length or spacing, can be associated with the stage of root development [23]. These changes in root development have been reported to correlate with tooth position relative to the occlusal plane [24]. Haavikko et al. (1970) [25] observed that tooth eruption typically coincides with a root that has reached approximately two-thirds of its full length. Based on this, Liversidge and Molleson (2017) [26] demonstrated that a tooth generally reaches the occlusal plane (functional eruption) when about three-quarters of the root is complete. Thus, when a tooth is observed near or at the occlusal plane, observers may be predisposed to assign a developmental stage consistent with the visible eruption, even if the root morphology alone indicates a less advanced stage.

This presents a methodological challenge, as features external to the tooth being assessed may influence stage assignment, thereby reducing reproducibility and introducing bias. By presenting the tooth within its full anatomical context, the examiner may integrate information that is correlated with development but not part of the diagnostic criteria of the Demirjian staging system [15]. This can increase both inter- and intra-observer discrepancies, particularly in borderline cases where distinctions between stages rely on subtle changes in apical shape, root elongation and crown-root proportions.

Cropping the image to isolate the tooth could be a useful solution to test the true influence of peripheral cues in tooth staging. By removing surrounding anatomical structures, cognitive bias may be reduced, as factors such as eruption level and proximity to the occlusal plane are no longer considered during assessment. It can also minimise distraction from neighbouring teeth and bone patterns allowing observers to focus solely on crown-root development. Conversely, removing this context might also deprive experienced observers of corroborating information; whether cropping reduces reproducibility, leaves it unchanged, or affects it differentially across tooth types is therefore an empirical question that the present study sought to address.

This study aimed to investigate whether cropping panoramic radiographs can influence developmental stage allocation considering one of the most widely used DAE staging systems.

## Materials and methods

### Data collection

This observational analytical cross-sectional study was conducted using 301 digital panoramic radiographs. All radiographs included in this retrospective study had been acquired digitally using a Vatech panoramic radiography unit (Vatech Co., Ltd., Hwaseong, Republic of Korea). The selected sample consisted of anonymized panoramic radiographs from subjects aged 7 to 14 years, including both males and females. The sample comprised 150 males and 151 females, with a mean age of 10.98 ± 2.26 years. The distribution of the sample by age and sex is presented in Table 1. No subjects were exposed to ionizing radiation solely for the purposes of this study. Anonymization was performed by extracting only the data relevant to the study, including the radiograph identification number, sex, date of birth, and date of radiographic acquisition.

**Table 1:** Age and sex distribution of the study sample

Age (years)	Male (*n*)	Female (*n*)	Total (*n*)
7	17	18	35
8	15	19	34
9	21	20	41
10	20	20	40
11	20	20	40
12	20	18	38
13	19	20	39
14	18	16	34
Total	150	151	301

The images were selected based on the presence of intact permanent teeth in the third quadrant. Radiographs exhibiting dental anomalies, supernumerary teeth, or any conditions potentially affecting normal dental development, such as tumors, fractures, pathological lesions, or a history of orthodontic treatment, were excluded. Subjects presenting with taurodontic teeth were excluded, as taurodontism is often associated with syndromic conditions that may affect normal dental development. Moreover, established DAE systems such as the Demirjian one are unreliable in such cases due to altered root morphology [27]. Furthermore, radiographs with poor image quality, including significant distortion or blurring that could impair accurate staging of tooth development, were also excluded. This study received ethical approval from the Health Research Ethics Committee of the Faculty of Medicine, Universitas Diponegoro (Approval No. 212/EC/KEPK/FK-UNDIP/VIII/2025). The study was carried out under the ethical standards laid down by the Declaration of Helsinki (Finland) and its later amendments [28].

### Inclusion of the mandibular third molar (FDI #38)

While the Demirjian staging system [15] was originally developed for the seven left permanent mandibular teeth, the left mandibular third molar (FDI #38) [29] was additionally evaluated. Staging the third molar often presents a greater challenge to observers due to its proximity to the mandibular ramus and the high frequency of angulation or crowding. It should be noted that within the 7–14-year age range studied, the third molar is expected to be present only in its earliest mineralization stages (approximately A–C).

### Radiograph cropping

**Fig. 1:**
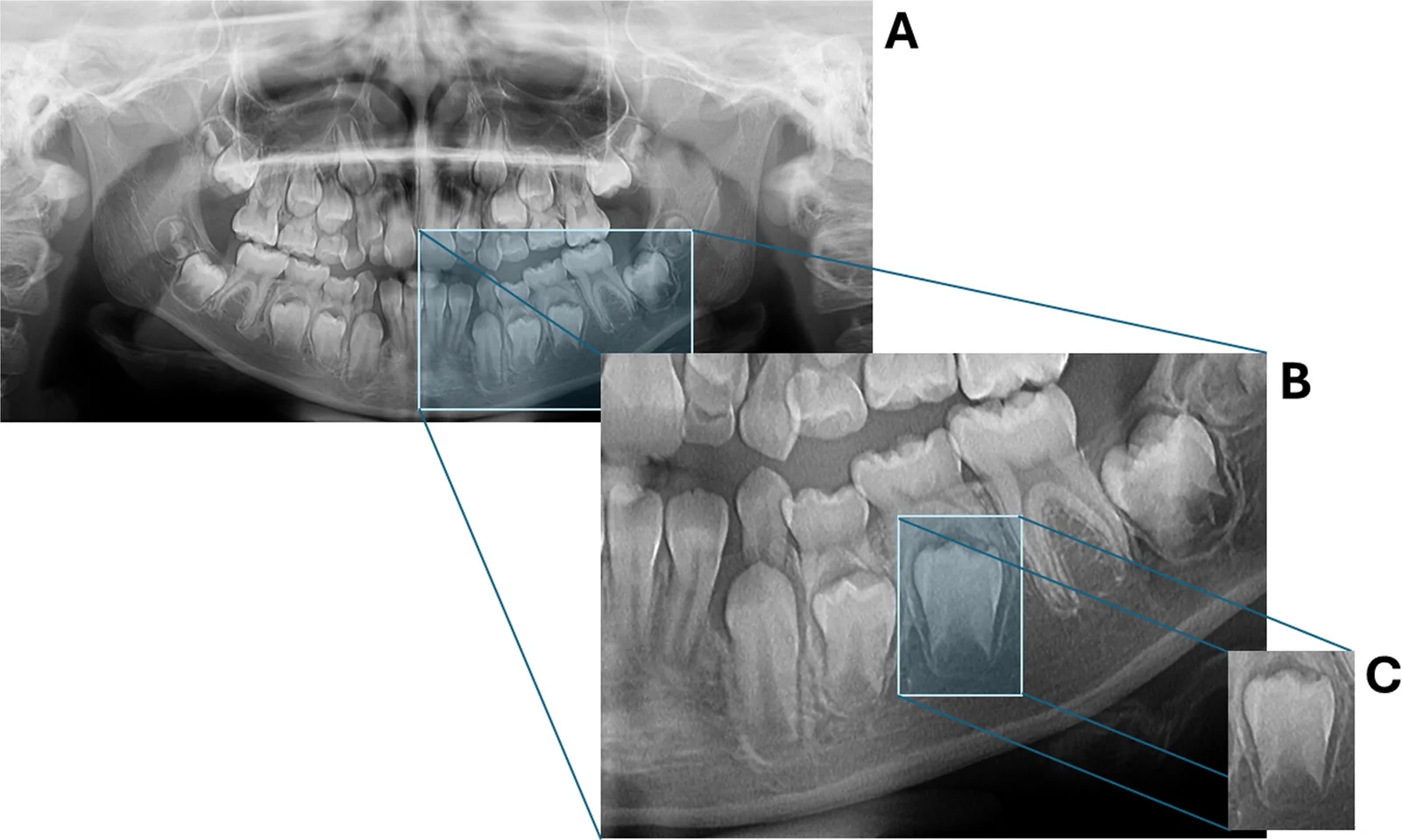
Illustration of the image selection process displaying an original radiograph (**A**), focus on the mandibular left permanent teeth (3rd quadrant, **B**) and the cropped region of interest (FDI 35, **C**)

To capture earlier developmental stages, younger subjects without any signs of third molar mineralization were also included; in such cases, only the seven left permanent mandibular teeth were staged. Within a 7–14-year cohort the available developmental stages are naturally skewed: the early-forming teeth (incisors and the first molar) are predominantly in the later stages (G–H), and the earliest stages (A–C) are essentially confined to the third molar (Table 2). Original images were duplicated according to the number of teeth to be staged, depending on the presence or absence of the left mandibular third molars, and then they were cropped to create several new images including all the seven or eight left mandibular teeth. During the cropping process, a margin was intentionally preserved beyond the lower boundary and above the upper boundary of the developing tooth whilst ensuring that the incisal/occlusal and apical information from adjacent teeth were not included in the cropped images (Fig. 1). This approach ensured that each image provided observers with a clear and unobstructed view of all relevant anatomical structures necessary for accurate assessment of the tooth’s developmental stage. During the cropping process, all seven or eight mandibular teeth from each radiograph were extracted, specifically teeth #31–#38 [29].

**Table 2:** Distribution of Demirjian developmental stages by tooth, as recorded by the primary observer (Observer B) at the first reading of the non-cropped images (one reading per radiograph; *n* = 301)

Tooth	A	B	C	D	E	F	G	H	NO	NA
FDI 31	0	0	0	0	0	2	20	268	10	1
FDI 32	0	0	0	0	1	29	44	203	23	1
FDI 33	0	0	0	0	45	101	56	86	13	0
FDI 34	0	0	0	4	64	89	57	85	1	1
FDI 35	0	0	1	16	74	106	53	48	1	2
FDI 36	0	0	0	0	0	8	88	204	0	1
FDI 37	0	0	6	41	80	89	58	26	0	1
FDI 38	42	71	42	15	27	4	1	0	0	99

*A-H* Demirjian’s stages and their distribution per tooth position, *NO* tooth present but unstageable, *NA* tooth not present (left blank)

For the second dataset, which included the cropped radiographs, the numbering system was organized as follows: if radiograph 1 was cropped, the resulting images were labeled sequentially according to the FDI tooth numbering system [29]. Thus, the cropped images were designated as 1.38, 1.37, 1.36, 1.35, 1.34, 1.33, 1.32, and 1.31, where “1” refers to the original panoramic radiograph and the subsequent two-digit numbers indicate the specific teeth extracted as cropped images. One of the co-authors, who did not participate as an observer in the radiographic staging (to preserve a blinded setup), performed both the image cropping and the creation of the working databases for analysis.

**Participants/observers, calibration and developmental stage allocation**.

Three qualified forensic odontologists (A, B and C), with at least eight years of experience in research and clinical DAE cases, independently performed staging of the two datasets. Calibration instructions were provided to each observer to ensure a standardized application of each method. The guidelines were as follows:

1. In cases of uncertainty regarding which stage to choose, the observer was instructed to select the lower stage.
2. For multi-rooted teeth exhibiting differing stages of development (e.g., the distal root at stage G and the mesial root at stage H), the lower stage was to be taken into consideration.
3. No changes were to be made to the opposite side if the tooth could not be observed.
4. When a tooth was present but could not be staged, observers were instructed to mark it as “NO” (tooth present but unstageable); teeth that were not present, or that could not be identified in each reading, were left unmarked and treated as “NA” (tooth not present).
5. Observers were allowed to use any image processing software to aid in the process, with a request to inform the project supervisors of the software chosen. Zoom-in and zoom-out functions were also allowed to facilitate more accurate evaluation of the radiographic images.

Observers were blinded to any radiographic metadata, including chronological age and sex to ensure minimal bias. Inter-observer reliability was assessed based on the initial evaluations, while intra-observer reliability was determined using repeated observations. To assess intra-observer agreement, all radiographs and cropped images were re-evaluated independently by each observer after a two-week washout period following the initial staging. All staging procedures were completed in January 2026.

### Statistical analysis

Two sampling quantities were defined for every agreement computation and were derived independently for each comparison rather than fixed a priori as a single anatomical tooth count. The denominator (N) represented the number of tooth observations that were jointly evaluable in a given comparison, i.e., recorded as present in every reading entering that specific analysis (both rounds for intra-observer agreement, and all three observers’ first readings for inter-observer agreement). If the tooth was not present (coded “NA”; e.g., absent, not yet formed, or not identifiable in a given reading), it was excluded from N for that comparison.

The numerator (n) represents the subset of N where all relevant observers assigned a valid Demirjian developmental stage (A–H). For intra-rater agreement, this required both rounds of assessment by the same observer to yield a valid stage. For inter-rater agreement, all three observers were required to have assigned a stage in their first-round assessments. Tooth observations recorded as present but unstageable (“NO”) were retained in N but excluded from n, as they do not contribute to the agreement calculation; teeth that were not present (“NA”) were excluded from N entirely.

It follows that N is expected to vary slightly across observers and between the cropped and non-cropped conditions. This variation does not represent a changing physical sample; it reflects the list-wise-complete nature of agreement statistics, which can be computed only on observations that are jointly available to all readings being compared. When a tooth was not present or could not be identified in a particular reading (coded “NA”), that observation carried no agreement information and was removed from the relevant comparison only, whereas teeth recorded as present but unstageable (“NO”) were retained in N and excluded only from n. Because observers occasionally differed in the small number of teeth they could confirm as present, and because the third molar (FDI #38) is biologically present in only a subset of this age cohort, small and expected differences in N arise between observers and conditions.

Intra-observer agreements were analysed between the first and second observation of three independent observers. Furthermore, the inter-observer agreement was assessed by comparing the first observation of each observer. All intra-observer agreements were analysed using Cohen’s kappa with quadratic weights. Inter-observer agreement among all three observers was assessed simultaneously using Round 1 staging under both conditions, quantified using Weighted Gwet’s AC2 [30] with ordinal weights. Gwet’s AC2 was selected for inter-observer analysis because it is less susceptible to the paradoxical reduction in kappa values that can occur when stage distributions are highly skewed or when the prevalence of certain categories is very high, and it can accommodate simultaneous comparison among three or more raters [30]. The 95% confidence intervals were calculated using the asymptotic standard error.

Agreement was interpreted using the Landis & Koch (1977) benchmarks, namely poor (< 0.00), slight (0.01-0.20), fair (0.21–0.40), moderate (0.41–0.6), substantial (0.61–0.80) and almost perfect (0.81–1) [31]. Changes were notated as Δ (cropped – non-cropped), where a positive value indicates higher agreement with cropped images, and a negative value indicates higher agreement with non-cropped. All analyses were performed using R version 4.5.2 (R Foundation for Statistical Computing, Vienna, Austria) with the psych package for Weighted Cohen’s Kappa computation.

## Results

All intra-observer agreements across the three observers reached above 0.81 with almost perfect agreement (Tables 3, 4 and 5). Varying degrees of agreement were observed among the cropped data, where only Observer A showed a slight improvement in Δ Kappa between the two imaging versions, and both Observers B and C showed a slight decrease in performance when evaluating cropped images (Δ −0.017 for Observer B; Δ −0.023 for Observer C). Even so, the differences were marginal.

**Table 3:** Intra-observer agreement from observer A

OBSERVER A					
Tooth Group	Non-Cropped		Cropped		Δ Kappa
	n/N (%)	Kappa [95% CI]	n/N (%)	Kappa [95% CI]	
All Teeth					
Overall	2080/2276 (91.4%)	0.977 [0.974; 0.981]	2077/2289 (90.7%)	0.982 [0.979; 0.985]	+ 0.005
Region					
Anterior	748/900 (83.1%)	0.946 [0.932; 0.960]	731/901 (81.1%)	0.958 [0.946; 0.971]	+ 0.012
Posterior	1332/1376 (96.8%)	0.977 [0.973; 0.981]	1346/1388 (97.0%)	0.982 [0.978; 0.985]	+ 0.005
Tooth Type					
Incisors	501/600 (83.5%)	0.910 [0.873; 0.947]	480/600 (80.0%)	0.897 [0.859; 0.936]	−0.013
Canine	247/300 (82.3%)	0.921 [0.894; 0.948]	251/301 (83.4%)	0.952 [0.932; 0.971]	+0.031
Premolars	579/600 (96.5%)	0.950 [0.939; 0.960]	572/599 (95.5%)	0.967 [0.958; 0.976]	+ 0.017
Molars	753/776 (97.0%)	0.984 [0.980; 0.988]	774/789 (98.1%)	0.985 [0.981; 0.989]	+ 0.001
Individual Teeth					
FDI 31	261/300 (87.0%)	0.836 [0.714; 0.957]	252/300 (84.0%)	0.880 [0.765; 0.996]	+ 0.044
FDI 32	240/300 (80.0%)	0.919 [0.880; 0.958]	228/300 (76.0%)	0.892 [0.849; 0.935]	−0.027
FDI 33	247/300 (82.3%)	0.921 [0.894; 0.948]	251/301 (83.4%)	0.952 [0.932; 0.971]	+ 0.031
FDI 34	285/300 (95.0%)	0.950 [0.934; 0.965]	280/300 (93.3%)	0.967 [0.953; 0.981]	+ 0.017
FDI 35	294/300 (98.0%)	0.947 [0.932; 0.962]	292/299 (97.7%)	0.965 [0.954; 0.976]	+ 0.018
FDI 36	299/300 (99.7%)	0.887 [0.840; 0.934]	298/299 (99.7%)	0.886 [0.823; 0.950]	−0.001
FDI 37	295/299 (98.7%)	0.952 [0.933; 0.972]	297/300 (99.0%)	0.951 [0.934; 0.969]	−0.001
FDI 38	159/177 (89.8%)	0.928 [0.901; 0.956]	179/190 (94.2%)	0.942 [0.919; 0.965]	+ 0.014

**Table 4:** Intra-observer agreement from observer B

OBSERVER B					
Tooth Group	Non-Cropped		Cropped		Δ Kappa
	n/N (%)	Kappa [95% CI]	n/N (%)	Kappa [95% CI]	
All Teeth					
Overall	2242/2296 (97.6%)	0.994 [0.992; 0.995]	2203/2301 (95.7%)	0.977 [0.969; 0.984]	−0.017
Region					
Anterior	847/899 (94.2%)	0.981 [0.972; 0.989]	812/901 (90.1%)	0.917 [0.865; 0.969]	−0.064
Posterior	1395/1397 (99.9%)	0.994 [0.993; 0.996]	1391/1400 (99.4%)	0.982 [0.978; 0.986]	−0.012
Tooth Type					
Incisors	562/599 (93.8%)	0.972 [0.953; 0.991]	526/600 (87.7%)	0.857 [0.768; 0.945]	−0.115
Canine	285/300 (95.0%)	0.971 [0.954; 0.988]	286/301 (95.0%)	0.940 [0.909; 0.971]	−0.031
Premolars	595/597 (99.7%)	0.986 [0.980; 0.992]	595/599 (99.3%)	0.964 [0.954; 0.974]	−0.022
Molars	800/800 (100.0%)	0.996 [0.994; 0.997]	796/801 (99.4%)	0.985 [0.980; 0.990]	−0.011
Individual Teeth					
FDI 31	288/300 (96.0%)	0.960 [0.904; 1.000]	268/300 (89.3%)	0.898 [0.828; 0.968]	−0.062
FDI 32	274/299 (91.6%)	0.971 [0.950; 0.993]	258/300 (86.0%)	0.837 [0.721; 0.952]	−0.134
FDI 33	285/300 (95.0%)	0.971 [0.954; 0.988]	286/301 (95.0%)	0.940 [0.909; 0.971]	−0.031
FDI 34	298/299 (99.7%)	0.983 [0.974; 0.992]	296/300 (98.7%)	0.966 [0.953; 0.978]	−0.017
FDI 35	297/298 (99.7%)	0.988 [0.980; 0.996]	299/299 (100.0%)	0.961 [0.945; 0.977]	−0.027
FDI 36	299/299 (100.0%)	0.939 [0.903; 0.975]	299/300 (99.7%)	0.924 [0.880; 0.969]	−0.015
FDI 37	299/299 (100.0%)	0.971 [0.960; 0.982]	300/300 (100.0%)	0.948 [0.925; 0.971]	−0.023
FDI 38	202/202 (100.0%)	1.000 [1.000; 1.000]	197/201 (98.0%)	0.938 [0.917; 0.959]	−0.062

**Table 5:** Intra-observer agreement from observer C

OBSERVER C					
Tooth Group	Non-Cropped		Cropped		Δ Kappa
	n/N (%)	Kappa [95% CI]	n/N (%)	Kappa [95% CI]	
All Teeth					
Overall	2231/2301 (97.0%)	0.997 [0.995; 0.998]	2189/2299 (95.2%)	0.974 [0.967; 0.982]	−0.023
Region					
Anterior	840/900 (93.3%)	0.996 [0.992; 0.999]	804/901 (89.2%)	0.916 [0.864; 0.968]	−0.08
Posterior	1391/1401 (99.3%)	0.996 [0.994; 0.997]	1385/1398 (99.1%)	0.978 [0.974; 0.983]	−0.018
Tooth Type					
Incisors	563/599 (94.0%)	0.991 [0.980; 1.000]	523/600 (87.2%)	0.849 [0.760; 0.939]	−0.142
Canine	277/301 (92.0%)	0.995 [0.990; 1.000]	281/301 (93.4%)	0.941 [0.911; 0.972]	−0.054
Premolars	595/600 (99.2%)	0.990 [0.984; 0.997]	592/599 (98.8%)	0.953 [0.942; 0.965]	−0.037
Molars	796/801 (99.4%)	0.997 [0.996; 0.998]	793/799 (99.2%)	0.983 [0.978; 0.988]	−0.014
Individual Teeth					
FDI 31	289/300 (96.3%)	1.000 [1.000; 1.000]	267/300 (89.0%)	0.856 [0.769; 0.943]	−0.144
FDI 32	274/299 (91.6%)	0.988 [0.973; 1.000]	256/300 (85.3%)	0.838 [0.722; 0.955]	−0.15
FDI 33	277/301 (92.0%)	0.995 [0.990; 1.000]	281/301 (93.4%)	0.941 [0.911; 0.972]	−0.054
FDI 34	298/300 (99.3%)	0.988 [0.977; 0.999]	295/300 (98.3%)	0.958 [0.944; 0.972]	−0.03
FDI 35	297/300 (99.0%)	0.993 [0.987; 0.999]	297/299 (99.3%)	0.946 [0.927; 0.966]	−0.047
FDI 36	299/301 (99.3%)	0.989 [0.983; 0.996]	299/300 (99.7%)	0.919 [0.873; 0.964]	−0.069
FDI 37	298/300 (99.3%)	0.989 [0.982; 0.996]	300/300 (100.0%)	0.942 [0.920; 0.964]	−0.047
FDI 38	198/200 (99.0%)	0.986 [0.978; 0.995]	194/199 (97.5%)	0.931 [0.908; 0.954]	−0.055

The most pronounced decreases were observed for individual anterior teeth. For example, Observer C showed a decrease from κ = 1.000 to 0.856 for FDI 31 (Δ − 0.144) and from κ = 0.988 to 0.838 for FDI 32 (Δ − 0.150) when using cropped images, while posterior teeth generally maintained high agreement values regardless of imaging format.

These marginal differences were also observed in the inter-observer agreement, where the choice between non-cropped and cropped images had a negligible impact on overall inter-observer agreement. The overall inter-observer agreement was nearly identical across modalities, with an AC2 of 0.985 (95% CI [0.984; 0.987]) for non-cropped and 0.986 (95% CI [0.985; 0.987]) for cropped images (Δ + 0.001), with consistently high values across regions, tooth types, and individual teeth (Table 6). However, the proportion of assessable radiographs was slightly lower for cropped images, with 91.7% of non-cropped images deemed assessable by all observers compared with 90.7% for cropped images.

**Table 6:** Inter-rater agreement between all observers

INTER-OBSERVER AGREEMENT (3 Observers)					
Tooth Group	Non-Cropped		Cropped		Δ AC2
	n/N (%)	AC2 [95% CI]	n/N (%)	AC2 [95% CI]	
All Teeth					
Overall	2097/2286 (91.7%)	0.985 [0.984; 0.987]	2079/2292 (90.7%)	0.986 [0.985; 0.987]	+ 0.001
Region					
Anterior	759/900 (84.3%)	0.994 [0.993; 0.995]	726/901 (80.6%)	0.994 [0.993; 0.995]	0
Posterior	1338/1386 (96.5%)	0.979 [0.977; 0.982]	1353/1391 (97.3%)	0.980 [0.978; 0.982]	+ 0.001
Tooth Type					
Incisors	509/599 (85.0%)	0.997 [0.996; 0.998]	472/600 (78.7%)	0.997 [0.996; 0.998]	0
Canine	250/301 (83.1%)	0.986 [0.982; 0.989]	254/301 (84.4%)	0.987 [0.983; 0.990]	+ 0.001
Premolars	584/599 (97.5%)	0.984 [0.981; 0.987]	576/599 (96.2%)	0.984 [0.981; 0.987]	0
Molars	754/787 (95.8%)	0.977 [0.973; 0.981]	777/792 (98.1%)	0.979 [0.976; 0.982]	+ 0.002
Individual Teeth					
FDI 31	265/300 (88.3%)	0.999 [0.998; 0.999]	247/300 (82.3%)	0.998 [0.997; 0.999]	−0.001
FDI 32	244/299 (81.6%)	0.994 [0.992; 0.997]	225/300 (75.0%)	0.996 [0.994; 0.998]	+ 0.002
FDI 33	250/301 (83.1%)	0.986 [0.982; 0.989]	254/301 (84.4%)	0.987 [0.983; 0.990]	+ 0.001
FDI 34	289/300 (96.3%)	0.985 [0.981; 0.989]	283/300 (94.3%)	0.986 [0.983; 0.990]	+ 0.001
FDI 35	295/299 (98.7%)	0.983 [0.979; 0.987]	293/299 (98.0%)	0.982 [0.978; 0.986]	−0.001
FDI 36	298/299 (99.7%)	0.997 [0.996; 0.998]	297/299 (99.3%)	0.997 [0.996; 0.998]	0
FDI 37	294/300 (98.0%)	0.972 [0.964; 0.979]	297/300 (99.0%)	0.972 [0.967; 0.977]	0
FDI 38	162/188 (86.2%)	0.965 [0.959; 0.972]	183/193 (94.8%)	0.975 [0.969; 0.980]	+ 0.01

## Discussion

This study investigated whether isolating teeth as regions of interest through cropping of panoramic radiographs influences the reproducibility of stage allocation when applying the Demirjian staging system [15]. DAE based on radiographic analysis is a procedure with clinical and forensic purposes [32, 33]. The findings indicated that cropping did not significantly influence staging agreement. Instead, it slightly but consistently reduced kappa values across most tooth groups, regions, and individual teeth. Nevertheless, the magnitude of these differences was small, and agreement remained within the range of almost perfect or substantial in both imaging conditions.

The Demirjian staging system was selected as the reference method for several reasons. First, it comprises a comparatively smaller number of developmental stages than other multi-stage DAE methods such as Moorrees’ [34] and Nolla’s [13], which renders it practical and straightforward to apply, and its relatively simple structure facilitates examiner training and calibration [35–37], thereby enhancing reproducibility across evaluators. Second, it has become the most widely used and extensively cited approach in the field [38], as confirmed by a recent bibliometric analysis [39]. Third, it has been validated across a wide variety of populations, providing a comparative basis for cross-national applications [38, 40, 41]. Although originally designed for the seven left mandibular permanent teeth, the method has since been extended to the third molars, broadening its relevance for age estimation in older adolescents and young adults [35, 42, 43].

Intra-observer agreement, assessed using quadratically weighted Cohen’s kappa, was consistently high across all three observers, with all values exceeding 0.81, corresponding to almost perfect agreement. These results confirm the strong reproducibility of the Demirjian staging system [15] when applied by experienced observers. This is consistent with a previous study demonstrating excellent intra- and inter-observer reliability of the system [15] when applied by experienced forensic experts [35]. It must be noted, however, that while Demirjian staging system [15] is reproducible, it has fewer stages than other consolidated systems, namely Moorrees’ and Nolla’s [13, 34]. As a drawback, fewer stages can be less descriptive toward the dental development process, especially in teeth with long formation time, such as third molars. This is an indication that staging-based third molar DAE could be better accomplished with other systems, such as Gleiser and Hunt’s [44] modified by Köhler [45] or even adaptations of Demirjian staging system [15] itself in addition to the other two previously mentioned.

Although one observer demonstrated a slight improvement when using regions of interest radiographs, the other two showed small decreases in agreement. This pattern suggests that cropping does not systematically improve intra-observer consistency. The reduction in agreement was more pronounced in anterior teeth, particularly incisors, where the largest decreases in weighted kappa values were observed. This pattern is also consistent with well-recognised limitations of panoramic imaging in the anterior region, where lower spatial resolution, geometric distortion, and superimposition within the narrow anterior focal trough can obscure the subtle root features on which incisor staging depends [18, 19]. In contrast, posterior teeth, especially molars, showed minimal variation between full and cropped conditions, maintaining consistently high agreement regardless of image format. This stability is likely explained in part by the limited number of developmental stages present in these teeth at the ages studied (predominantly G–H; Table 2), in addition to their more distinctive root morphology, which together allow reliable staging even in the absence of surrounding anatomical cues. Inter-observer agreement, assessed using weighted Gwet’s AC2 [30], remained remarkably stable across image formats. The overall agreement was nearly identical between non-cropped and cropped images, with only a negligible difference between conditions. The consistently high AC2 values observed in both conditions indicate that agreement between observers was robust and not influenced by the choice of image presentation. These findings suggest that, while cropping may slightly affect individual assessment, it does not introduce overall inconsistency among experienced observers. In practice, however, every effort to improve DAE procedures is valuable in promoting more reliable performance. In this context, the academic community should consider providing more descriptive rationale for tooth developmental stages. The currently available staging systems [13, 15, 34, 44–47] either focus on (1) predictive remaining root formation, labeling stages as “¼ or ¾” of root, and (2) proportional-morphological explanations, where the length of root and crown are compared or the shape of dental anatomy is addressed. While the former can be uncertain because knowledge of full tooth development is predicted (the expert observes and knows the radiographic representation of tooth formation and predicts how much will remain until apical closure), the latter provides a rationale more based on the present and available amount of tooth parts. The Demirjian staging system [15] is an example of the latter. The present findings suggest that its application could be enhanced by increasing the granularity of the developmental stages, as previously discussed, and by incorporating more detailed descriptors of the peripheral region of the assessed tooth, particularly for third molar staging [48].

Importantly, inter-observer agreement was almost identical between the two conditions, so the present data do not provide statistical evidence that adjacent anatomical context stabilises stage allocation. The small, non-significant intra-observer decreases observed with cropping are compatible with — but do not demonstrate — a supportive role of contextual information, given that stage allocation is the most subjective step in DAE [35].

Observer experience is also an important factor. In this study, three trained forensic odontologists independently evaluated the images. Their level of expertise likely contributed to the high agreement observed in both conditions and may have mitigated potential contextual bias. It is plausible that less experienced observers would be more susceptible to contextual influence, and that cropping might have a different effect in such populations. Future studies should therefore evaluate the influence of observer experience on the impact of image presentation.

Although there is increasing research interest in artificial intelligence, and consequently the possibility of automating decision-making, human observers remain central in this process, especially when computer-based learning is trained from databases that were originally set after human interaction with dental staging. The present findings reinforce the importance of understanding how human observers and specialists interact with radiographic information, including the combined role of morphological criteria and contextual anatomical cues.

### Limitations

Several limitations should be considered when interpreting these findings. The distribution of developmental stages was skewed towards the later stages for the early-forming teeth: at 7–14 years, the mandibular incisors and first molar were almost exclusively in stages G–H, with stages A–C essentially absent, whereas the earliest stages were represented almost only by the third molar (Table 2). Because agreement coefficients tend to be higher when fewer competing stages are available, the very high reproducibility recorded for these teeth partly reflects this limited stage variability rather than method robustness alone, and the results may not generalize to samples spanning the full A–H range. Furthermore, all three observers were highly experienced forensic odontologists; the potential supportive effect of contextual anatomy may differ for less-experienced observers and warrants dedicated study.

## Conclusion

Within an experienced-observer setting, cropping panoramic radiographs to isolate individual teeth did not meaningfully reduce the reproducibility of Demirjian stage allocation. Intra-observer agreement remained almost perfect under both conditions, and inter-observer agreement was identical between non-cropped and cropped images, indicating that the presence or absence of surrounding anatomical context does not affect staging consistency among trained observers. The only consistent, though small, decreases with cropping were observed in the anterior teeth. These results indicate that both full-field and cropped presentations are acceptable for expert Demirjian staging, and that cropping can be applied without compromising reliability. Whether adjacent anatomical context provides measurable support for less-experienced observers, and whether more descriptive staging criteria would further improve anterior-tooth reproducibility needs dedicated future research.

## Data Availability

The datasets analysed during the current study are not publicly available due to ethical and privacy restrictions but are available from the corresponding author on reasonable request.
